# Bandgap Engineering through Topological and Strain-Induced Changes in Tetragraphene

**DOI:** 10.1021/acsomega.5c04245

**Published:** 2025-07-24

**Authors:** Wjefferson Henrique da Silva Brandão, Eduardo Costa Girão, Marcelo Lopes Pereira, Andrea Latgé

**Affiliations:** † Institute of Physics, 133643 Fluminense Federal University, Niterói, Rio de Janeiro 24210-340, Brazil; ‡ Department of Physics, 67823 Federal University of Piauí, Teresina, Piauí 64049-550, Brazil; § University of Brasília, College of Technology, Department of Electrical Engineering, Brasília 70910-900, Brazil

## Abstract

The ability to modulate electronic properties in low-dimensional carbon materials is fundamental to developing next-generation flexible electronics. In this work, we perform a comprehensive first-principles investigation of tetragraphene nanotubes (TGNTs), exploring the interplay between curvature-induced topology and uniaxial strain. Two chiral families are examined: zigzag-like (*n*, 0) and armchair-like (0, *m*) configurations. Our results show that all TGNTs remain semiconducting upon rolling, with direct band gaps at the Γ point. We show that (*n*, 0) TGNTs undergo a semiconductor-to-metal transition under strain, while preserving the sp^2^–sp^3^ hybridization, a phenomenon not previously reported for this class of materials. The nanotubes exhibit high Young’s modulus values and direction-dependent fracture patterns, with a strong correlation between structural anisotropy and mechanical performance. These findings reveal the potential of TGNTs as versatile platforms for strain-tunable optoelectronic applications and highlight the importance of topological and mechanical control in the engineering of functional nanocarbon systems.

## Graphical abstract

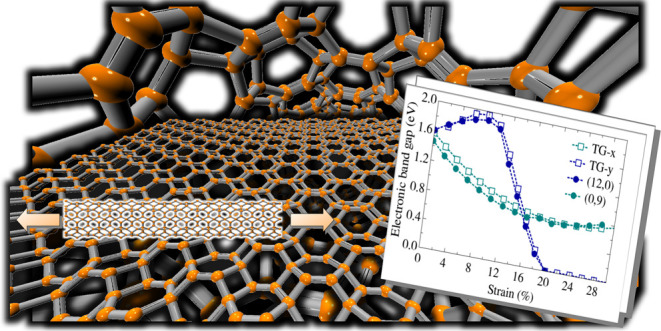

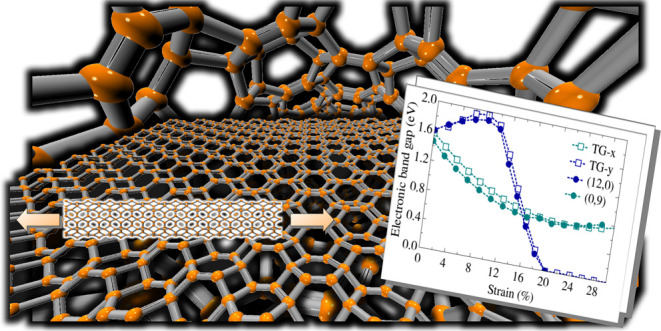

## 1 Introduction

Groundbreaking experiments and subsequent studies have revealed remarkable physical and chemical properties of graphene^1–5^ which has stimulated a broad scientific community to focus on the study of new two-dimensional (2D) carbon-based materials. The search for low-dimensional materials with tailored properties, including atomic sheets (2D) and nanotubes/nanoribbons (quasi-1D), has been driven by their potential applications in nanoelectronics, optoelectronics, and advanced charge-transport devices.^6–8^ Beyond expanding the potential of carbon systems for applications on the nanoscale, the theoretical proposal of atomic arrangements plays a crucial role in identifying promising materials before their experimental synthesis^9^ differently from the conventional honeycomb lattice of graphene. Notable examples include fully *sp* ^2^ (or possibly mainly *sp* ^2^) systems such as the biphenylene (BPN)^10–12^ and the fullerene (2D-C_60_) networks.^13^ Also important are systems containing a mixture of carbon atoms with distinct hybridization states, like γ-graphyne (γ-GY)^14–16^ where *sp* and *sp* ^2^ atoms coexist. These structures were initially proposed theoretically and subsequently synthesized in recent years, highlighting the relevance of the proposal of hypothetical nanostructures by computational simulations. While simulations can be used to interpret experimental outcomes, they can also stimulate and guide experimental research toward functional materials.

Other structures exhibiting mixed *sp* ^2^ and *sp* ^3^ hybridizations have garnered significant interest due to their improved structural flexibility and tunable band gap. One of the first examples was pentagraphene (PG)^17^ a structure that does not feature a hexagonal lattice (as suggested by the term “graphene”), but that contains exclusively pentagonal rings in a trilayer setup. While a structure fully formed by pentagons is impossible to draw for *sp* ^2^-like carbon, due to simple geometry constraints^18^ it is the presence of *sp* ^3^ carbon that allows the formation of a two-dimensional carbon Cairo-like tilling. PG has a wide gap that can be further tuned by defects^19^ or nanoribbon^20^ and nanotube setups.^21^ Other mixed *sp* ^2^-*sp* ^3^ nanocarbons have followed PG as the subject of theoretical proposals, such as penta-hexa-graphene^22^ and corresponding nanotubes^23^ and nanoribbons.^24^ Tetrahexcarbon^25^ or Tetragraphene (TG)^26^ is another example in which 4- and 6-membered rings fill the plane with a ratio of two *sp* ^2^ carbon to each *sp* ^3^ site. It was first proposed as a derivative of PG, through the insertion of a Stone–Wales transformation applied to PG *sp* ^2^ dimers.^25^ Its structure consists of one tetragonal and one hexagonal ring per primitive cell, arranged in a trilayer setup (as PG), where lower and upper *sp* ^2^ carbon layers are held together by a central plane of *sp* ^3^ atoms. This configuration results in a direct band gap of approximately 3.70 eV, as determined by density functional theory (DFT) calculations that employ the Heyd-Scuseria-Ernzerhof (HSE06) hybrid functional.^25^ Furthermore, TG exhibits high in-plane electronic mobility, making it a promising candidate for nanoelectronic applications, particularly in transistors, optoelectronic devices, and flexible electronic systems, where high carrier mobility and tunable electronic properties are essential.^27–29^

The ability to engineer the band gap of a material is fundamental for electronic and optoelectronic applications.^30^ Several strategies, such as quantum confinement (through nanotube/nanoribbon setups), chemical functionalization, and mechanical strain, are widely used to tailor the electronic properties of 2D materials.^31–33^ In the case of TG, previous studies have shown that its electronic structure can be modulated by hydrogenation, fluorination, and the formation of nanoribbons (TGNR).^34–36^ Previous investigations have shown that the transformation of TG sheets into TGNTs is an intriguing approach to exploring the effects of curvature on their electronic properties, which strongly depend on tube diameter and chirality. Zigzag-oriented TG nanotubes (TGNTs-z), for instance, exhibit significant curvature effects, reducing the effective mass of electrons and enhancing the mobility of charge carriers.^37^ Here, we have performed DFT calculations of tetragraphene membranes and tubes in two main families, *(n ,0)* and *(0, m)*, which agree with the first-principles results reported. We have focused on investigating the possibilities of driving electronic and mechanical changes by topological and strain-induced effects, as the interplay between nanotube formation and mechanical strain has not yet been systematically explored for the TG case. In particular, no previous study has reported a metal–insulator transition in TG-based systems under extreme strain conditions.

In this work, we explore the structural, electronic, and mechanical properties of TGNTs, with a focus on the combined effects of curvature and strain-induced transformations. It is important to mention that CNTs have been extensively investigated in the past under different spatial geometrical configurations, functionalization processes, and mechanical and radial deformations. As is well known, CNTs are very sensitive to curvature and chirality, changing from semiconducting to metallic electronic nature, depending upon their intrinsic structural aspects such as chirality and radii. While graphene is a gapless material, TG is a semiconducting material in its pristine form, favoring it for many electro-optical applications in comparison to graphene. Furthermore, TGNTs are always semiconducting in their pristine form, so that obtaining semiconducting TGNTs in eventual synthesis routes does not need to be constrained to grown systems with specific chiralities or diameters. Another important difference between TGNTs and CNTs is that wide TGNTs are not expected to be so easily deformed from their respective pristine circular configurations (as occurs for CNTs), since TGNT walls are a trilayer structure and the graphitic wall of CNTs is a single-atom-thick structure. On the other hand, nanotubes based on porous 2D nanocarbons (such as graphynes) are much easier to deform than TGNTs and CNTs.^38,39^ Here, we evaluated structural stability and mechanical properties of tetragraphene materials, as well as their modulation through topological transformations (nanotubes) and uniaxial strain, using DFT-based calculations. Furthermore, we analyze the mechanical properties as a function of chirality and nanotube diameter. Our findings reveal that TG transitions from a semiconducting to a metallic state can occur under an applied uniaxial strain of approximately 20%, depending on the tube diameter and chirality. These results offer insights into strain-driven phase transitions in low-dimensional carbon materials, providing new perspectives for strain-engineered nanoelectronics.

## 2 Methodology

A well-established methodological approach was employed to investigate the structural, electronic, and mechanical properties of the TG-based systems studied in this work. This process encompasses modeling 2D TG and its derived nanotubes, evaluating their stability, and computationally analyzing their properties under mechanical deformation. The methodology also includes a detailed description of the parameters and techniques used in the simulations, particularly within the DFT framework.

### 2.1 System’s Modeling

To model the TGNTs investigated in this work, we initially consider the 2D TG system, as shown in Figure 1a. The two-dimensional structure is constructed from a conventional rectangular unit cell defined by the orthogonal lattice vectors ${\mathrm{a⃗}}_{1}$ and ${\mathrm{a⃗}}_{2}$ , containing 12 carbon atoms. The primitive cell of TG is centered rectangular, and accommodates six atoms (4/2 carbons with *sp* ^2^/*sp* ^3^-like hybridization), with an angle of ∼73.15° between the primitive lattice vectors.^26^ However, the doubled (rectangular) cell with 12 atoms shown in Figure 1a is more convenient to work with^25^ especially for the representation of electronic states over the reciprocal space. In general only two types of atoms can be distinguished in TG, namely the sites denoted as C_1_ and C_2_, as shown in Figure 1a. The C_1_ atoms exhibit *sp* ^2^ hybridization, forming bonds with another C_1_ atom and two C_2_ atoms. In contrast, C_2_ atoms are *sp* ^3^-hybridized and bind exclusively to C_1_ atoms, forming four bonds as expected for *sp* ^3^ configurations. There are also only two types of bonds, which we will refer to as *d*_11_ (between two C_1_ atoms) and *d*_12_ (between a C_1_ and a C_2_ atom).

**Figure 1:**
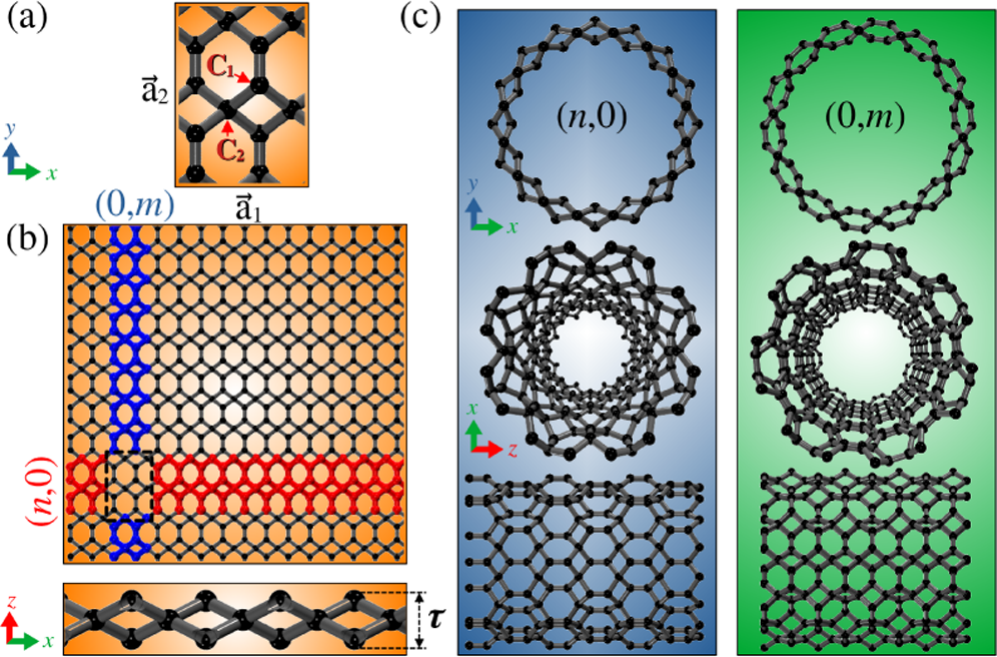
Schematic illustration of the construction of TGNTs from the TG sheet. (a) The unit cell of TG contains two types of carbon atoms. The orthorhombic lattice vectors ${\mathrm{a⃗}}_{1}$ and ${\mathrm{a⃗}}_{2}$ are also indicated. (b) Supercell of the TG sheet, highlighting the two rolling directions considered: zigzag-like *(n, 0)* (red) and armchair-like *(0, m)* (blue). The bottom panel shows the lateral view of the buckled TG structure, with τ representing the buckling amplitude. (c) Example structures of TGNTs obtained by rolling the TG sheet along two distinct directions. For each chirality, orthographic front view, perspective front view, and lateral view are presented.

Moreover, the presence of *sp* ^3^ hybridized atoms induces out-of-plane distortions, resulting in a buckled structure (or trilayer-like). The buckling, denoted by τ, is quantified as the absolute difference of the maximum and minimum out-of-plane coordinates of the carbon atoms, and it is depicted in the lower panel of Figure 1b. In addition, Figure 1b presents a supercell of the two-dimensional TG sheet. TG can also be viewed as a trilayer structure, with upper and lower layers formed by dimers of C_1_, while a central layer of C_2_ atoms connects the whole system together. Compared with PG, TG has a clear anisotropy verified from a simple visual inspection of its atomic structure. This is because the upper and lower C_1_–C_1_ dimers are aligned to the same crystallographic direction, while these two sets are aligned to directions orthogonal to each other in PG. This makes us expect stronger differences in the physical properties for TG tubes with different chiralities.

Since our objective is to construct TGNTs, the theoretical model consists of cutting the 2D TG sheet into multiples of its unit cell along the ${\mathrm{a⃗}}_{1}$ or ${\mathrm{a⃗}}_{2}$ vectors (analogous to nanoribbons), which is further rolled-up to form the nanotube geometry. The two cutting directions considered are highlighted in Figure 1b: the red line indicates cutting along the *x*-direction ( ${\mathrm{a⃗}}_{1}$ vector), while the blue line corresponds to the cutting along the *y*-direction ( ${\mathrm{a⃗}}_{2}$ vector). To rigorously describe this construction, we define the chiral vector as ${\mathrm{C⃗}}_{h}=n{\mathrm{a⃗}}_{1}+m{\mathrm{a⃗}}_{2}$ , where *n* and *m* are integers. The length of ${\mathrm{C⃗}}_{h}$ determines the circumference of the resulting nanotube. Consequently, the diameter (prerelaxation) is given by the expression $D\left(n,m\right)=\left|{\mathrm{C⃗}}_{h}\right|/\pi$ . It is important to note that this corresponds to an average diameter, since the planar TG structure is not a one-atom-thick sheet.

Given the quasi-one-dimensional nature of nanotube structures, it is necessary to determine the periodic length along the longitudinal axis. To ensure translational symmetry, we define the translational vector $\mathrm{T⃗}=p{\mathrm{a⃗}}_{1}+q{\mathrm{a⃗}}_{2}$ , which must satisfy ${\mathrm{C⃗}}_{h}\cdot \mathrm{T⃗}=0$ , implying that ${\mathrm{C⃗}}_{h}$ and T⃗ are orthogonal. This condition leads to the relation $np\left|{\mathrm{a⃗}}_{1}{|}^{2}+mq\right|{\mathrm{a⃗}}_{2}{|}^{2}=0$ . Thus, to satisfy ${\mathrm{C⃗}}_{h}⊥\mathrm{T⃗}$ for nonzero *n* and *m*, the condition $p/q=-\left(m/n\right)\cdot \left(|{\mathrm{a⃗}}_{2}{|}^{2}/|{\mathrm{a⃗}}_{1}{|}^{2}\right)$ is required. In practice, fulfilling this condition for generic (*n,m*) chiralities requires relatively large values of *p* and *q* due to the $\left|{\mathrm{a⃗}}_{2}{|}^{2}/\right|{\mathrm{a⃗}}_{1}{|}^{2}$ ration, resulting in nanotubes with hundreds of atoms, making DFT simulations computationally prohibitive.

Therefore, in this work, we restrict our study to nanotubes whose chiral vectors are aligned with the *x* or *y* axes shown in Figure 1. Specifically, when *m* = 0, the nanotubes are classified as *(n,0)*-TGNTs, with diameters given by $D\left(n,0\right)=n\left|{\mathrm{a⃗}}_{1}\right|/\pi$ . In this case, *p* = 0 and the translational vector reduces to $\mathrm{T⃗}=q{\mathrm{a⃗}}_{2}$ . Setting *q* = 1 defines the primitive cell of the nanotube, with periodic length equal to $\left|{\mathrm{a⃗}}_{2}\right|$ .

Similarly, when *n* = 0, the nanotubes correspond to *(0, m)*-TGNTs, with diameters $D\left(0,m\right)=m\left|{\mathrm{a⃗}}_{2}\right|/\pi$ , and translational vector $\mathrm{T⃗}=p{\mathrm{a⃗}}_{1}$ , with *p* = 1, implying a primitive cell length of $\left|{\mathrm{a⃗}}_{1}\right|$ . Figure 1c presents orthographic and perspective front views, as well as side views, of representative nanotubes with *(n, 0)* and *(0, m)* chiralities. Due to their structural similarity with graphene, we use an analogy with the honeycomb lattice and call these setups zigzag-like and armchair-like configurations, respectively. To systematically investigate the properties of TGNTs with diameters up to approximately 20 Å, we varied *n* from 3 to 10 for the *(n, 0)* family, and *m* from 2 to 14 for the *(0, m)* family, ensuring a broad sampling of small- to medium-diameter nanotubes.

### 2.2 Computational Framework

The structural, mechanical, and electronic properties of TG and its rolled-up nanotube counterparts (TGNTs) were investigated using first-principles calculations within the DFT framework, as implemented in the (SIESTA) code.^40^ The exchange-correlation energy was treated using the Perdew–Burke–Ernzerhof (PBE) formulation of the generalized gradient approximation (GGA).^41^ Electron–ion interactions were described using the Troullier-Martins norm-conserving pseudopotentials of the.^42^ At the same time, the Kohn–Sham orbitals were expanded in a double-ζ basis set with an additional polarization function (DZP), for which an energy shift of 0.05 eV was considered in their definition. A real-space integration grid with a mesh cutoff of 400 Ry was adopted to ensure convergence of the total energy and forces.^43^

All structures were optimized until the maximum residual atomic forces were lower than 10^–3^ eV/Å, with a self-consistent field (SCF) energy convergence criterion set to 10^–4^ eV. For TGNTs, periodic boundary conditions (PBC) were imposed along the longitudinal direction, with vacuum regions of 40 Å along the two other orthogonal directions to suppress interactions between periodic images. In the case of the TG sheet, PBC was applied within the two in-plane directions, while a vacuum spacing of 40 Å was introduced along the orthogonal direction. The Brillouin zone sampling was carried out using the Monkhorst–Pack scheme^44^ with 1 × 1 × 30 *k*-points for *(0, m)* TGNTs, 1 × 1 × 22 *k*-points for *(n*, *0)* TGNTs, and 30 × 22 × 1 *k*-points for the TG.

The uniaxial tensile strain was applied incrementally in steps of 1%, starting from the relaxed configurations, and the systems were deformed until structural failure was observed. For TGNTs, the strain was applied along the tube’s longitudinal direction, and due to their tubular topology, the radial direction was free to relax, allowing natural Poisson contraction or expansion. In contrast, the uniaxial strain was applied independently along the planar TG sheet’s *x* and *y* directions. In each case, the strained lattice vector was kept fixed while the perpendicular lattice vector and all atomic positions were allowed to relax, ensuring proper mechanical equilibrium under strain.

Stress values were extracted directly along the strained direction at each deformation step. Stress–strain curves were constructed accordingly, and Young’s modulus (*Y*) was determined from the initial linear regime (strain up to 1%) using a least-squares linear fitting procedure. The ultimate tensile strength and the corresponding fracture strain were identified as the maximum stress and the associated strain in the mechanical response.

Electronic properties were also evaluated for selected strained configurations. For representative TGNTs, band structures were calculated after full mechanical relaxation at each 1% strain step up to the fracture threshold, allowing a detailed investigation of strain-induced gap modulation. Additionally, unstrained TGNTs with various diameters were analyzed to assess curvature effects on the electronic band structure. The Brillouin zone for nanotube systems was sampled along the high symmetry line *r*(0.0, 0.0, 0.0) → *X*(0.5, 0.0, 0.0) [r­(0.0, 0.0, 0.0) → *Y*(0.0, 0.5, 0.0)] for *(0,m)* [(*n,0*)] tubes, corresponding to the periodic longitudinal direction.

For the planar TG sheet, the electronic band structure was computed along the high-symmetry path r­(0.0, 0.0, 0.0) → *X*(0.5, 0.0, 0.0) → *M*(0.5, 0.5, 0.0) → *Y*(0.0, 0.5, 0.0) in the rectangular Brillouin zone. In all cases, the energy scale was shifted so that the Fermi level was aligned to *E* = 0. This methodology provided a consistent framework for analyzing the evolution of mechanical and electronic properties under strain and curvature, with particular emphasis on band gap engineering in TG and TGNT systems.

## 3 Results and Discussion

### 3.1 Structural and Energetic Stability

Based on the modeling described in the previous section, we initially optimized the TG unit cell. The lattice vectors obtained for the rectangular cell have $\left|{\mathrm{a⃗}}_{1}\right|=4.56$ Å and $\left|{\mathrm{a⃗}}_{2}\right|=6.16$ Å, with bond lengths of 1.36 Å for (*d*_11_) C_1_–C_1_ bonds and 1.55 Å for (*d*_12_) C_1_–C_2_ connections. The buckling parameter was found to be τ = 1.17 Å, in agreement with previous literature.^25^ This unit cell was employed as the initial geometry for constructing 21 TGNTs, with *(n,0)* and *(0,m)* chiralities, and average diameters ranging from approximately 4.3 Å to 20.4 Å. The chirality indices varied from 3≤n≤14 and 2≤m≤10 . The number of atoms per unit cell was given by 12­(*n + m*), resulting in 36 to 168 atoms for *(n,0)* and 24 to 120 atoms for *(0,m)* configurations.

After structural optimization, the nanotubes exhibited the diameters and longitudinal unit cell lengths shown in Table 1. It is evident that smaller-diameter nanotubes experience noticeable longitudinal elongation. This is attributed to the intrinsic buckled structure of TG. Upon rolling, the out-of-plane atoms approach each other at the inner side of the tube, inducing geometric rearrangement and slight stretching to relieve internal stress. For instance, the (3,0)- and (0,2)-TGNT exhibit elongations of approximately 2.3% and 5.0%, respectively, relative to the 2D unit cell length. At these levels, the relaxed geometries deviate significantly from the typical TGNT pattern, leading to strong structural distortions. Therefore, in the following analyses, we restrict the discussion to nanotubes with *n* > 3 and *m* > 2.

**Table 1:** Optimized Structural Parameters of the Tetragraphene Nanotubes

*(n,0)*	*D* (Å)	*L* (Å)	*(0,m)*	*D* (Å)	*L* (Å)
(3,0)	4.57	6.30	(0,2)	4.25	4.79
(4,0)	5.93	6.24	(0,3)	6.07	4.66
(5,0)	7.34	6.21	(0,4)	7.98	4.62
(6,0)	8.77	6.20	(0,5)	9.90	4.60
(7,0)	10.22	6.19	(0,6)	11.85	4.59
(8,0)	11.66	6.18	(0,7)	13.78	4.58
(9,0)	13.11	6.18	(0,8)	15.74	4.58
(10,0)	14.58	6.18	(0,9)	17.69	4.58
(11,0)	16.01	6.17	(0,10)	19.66	4.57
(12,0)	17.47	6.17			
(13,0)	18.91	6.17			
(14,0)	20.36	6.17			

For the remaining nanotubes, the longitudinal unit cell length gradually stabilizes with increasing diameter, converging to approximately 6.17 Å for the *(n,0)* family and 4.57 Å for the *(0,m)* family. These values represent a residual elongation of about 1.0% compared to the 2D TG sheet, reflecting the diminishing impact of curvature for diameters beyond 12 Å. We observe that the *L* values gradually converge to the $\left|{\mathrm{a⃗}}_{1}\right|/\left|{\mathrm{a⃗}}_{2}\right|$ value from the 2D counterpart for large-diameter (0,*m*)/(*n*,0) structures, so that for significantly larger tubes (computationally prohibitive under the DFT framework), these lengths are expected to match the original TG lattice constants.

Moreover, visual inspection of the optimized structures confirms the preservation of the characteristic TG motifs-alternating tetragonal and hexagonal rings-across all chiralities. For smaller-diameter tubes, more pronounced angular distortions are observed, especially in the tetragonal units (as we will show below), indicating higher local strain. Nevertheless, the sp^3^ hybridization of the C_2_ atoms remains intact, and the buckling parameter τ fluctuates by less than 0.1 Å across the series. These findings indicate that the intrinsic buckled topology of TG is structurally preserved even under substantial curvature, confirming its mechanical resilience and geometric robustness in cylindrical configurations. However, curvature breaks the symmetry of the *d*_11_ and *d*_22_ bonds relative to the inner and outer *sp* ^2^ sublayers of the TGNT. As a general trend, the nanotube bond lengths involving atoms from the inner (outer) wall become shorter (longer) than the corresponding bonds from 2D TG. As mentioned before, the largest deviations in the bond length profile occur for the narrowest (4,0) and (0,3) nanotubes. We found 1.323 Å for the length of the inner *d*_11_ bond in (0,3), while this value varies from 1.322 Å to 1.344 Å for the other *(0,m)* tubes. This is shown by the green line with squares in Figure 2a, where we note that the internal *d*_11_ bond length gradually approaches the corresponding value of the 2D system as we increase tube diameter in the *(0,m)* cases. As seen from the green line with triangles in Figure 2a, the outer *d*_11_ bond lengths also approach the corresponding 2D value for wider *(0,m)* tubes. Similar trends are observed when we compare the inner and outer *d*_12_ bonds for *(0,m)* TGNTs, as seen from the green lines in the plot from Figure 2b. Similar results are shown by *(n,0)* systems, as shown by the blue color lines in Figure 2(a,b), even though the variation range for *d*_11_ and *d*_12_ is much broader for *(0,m)* tubes, as their C_1_–C_1_ dimers lie orthogonal to the tube’s periodic direction. The variation of the nanotubes bond-length profiles relative to 2D TG is also projected over the TG lattice and represented by a colormap in Figure 2c. The same is also visualized by color plots for the bond angles in Figure 2d. These results illustrate how the local strain (especially in the tetragonal units), associated with large bond length and angle variations (relative to the parent 2D sheet), is released as we move from narrow to wide tubes.

**Figure 2:**
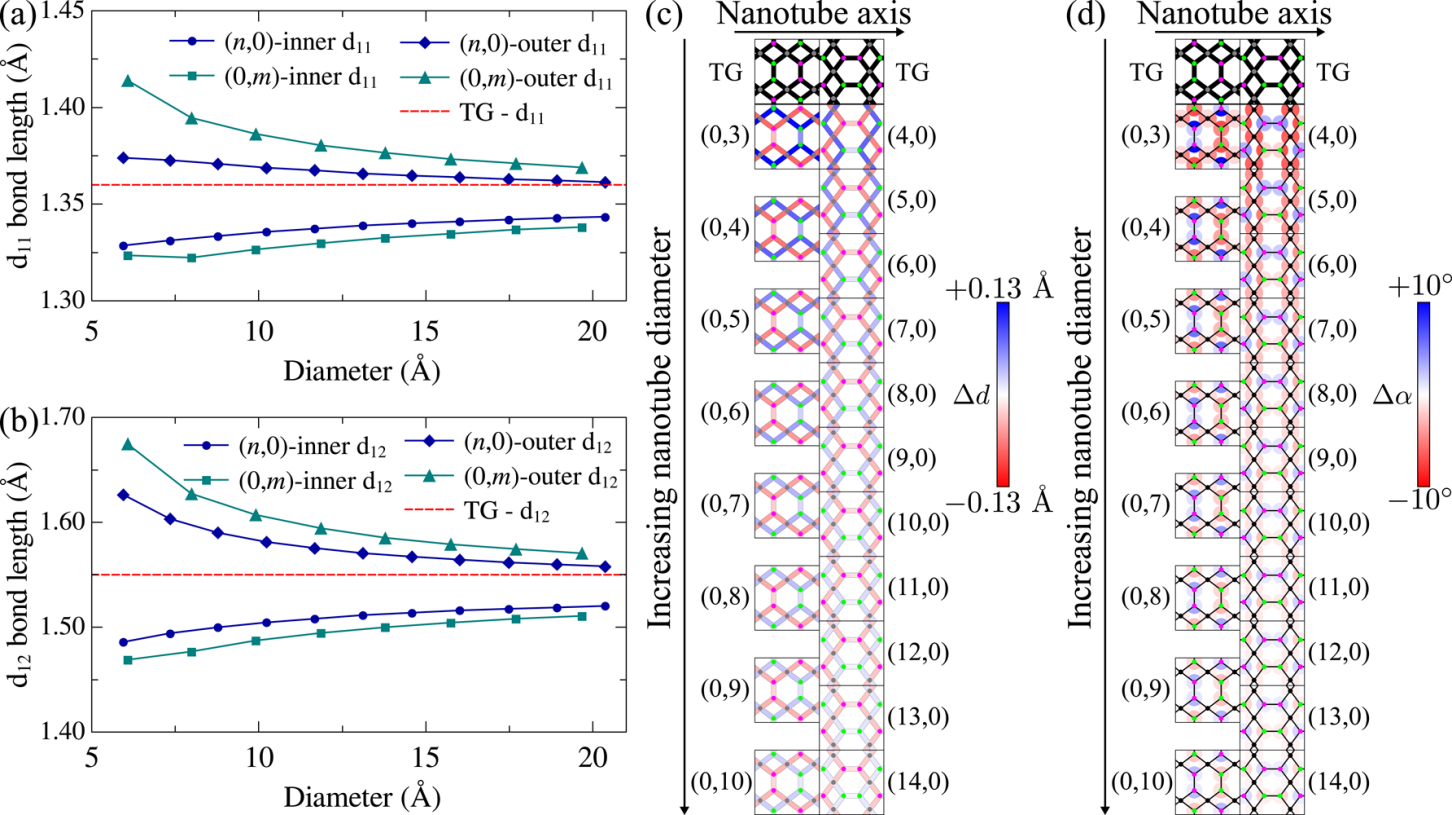
(a) Inner and outer *d*_11_ bond lengths for the *(n,0)* (blue lines) and *(0,m)* (green lines) TGNTs. (b) Same as (a), but for the *d*_12_ bond lengths. (c) Colormap for the (c) bond-length and (d) angle variations for the nanotube connections relative to the bond-length and angle profiles in the 2D TG counterpart. The green/purple atoms represent C_1_ sites in the outer/inner wall of the tube, while gray atoms correspond to C_2_ sites.

The energetic stability of TGNTs was further assessed by evaluating the curvature energy *E*_*c*_, defined as

1 $$E_{c}=\frac{E_{NT}}{N_{NT}}-\frac{E_{2D}}{N_{2D}}$$

where *E*_*NT*_ and *N*_*NT*_ denote the total energy and number of atoms in the nanotube, while *E*_*2D*_ and *N*_*2D*_ correspond to the energy and number of atoms in the planar TG reference. This quantity compares the energy per atom in a nanotube with the corresponding value in the 2D parent system. Figure 3 shows *E*_*c*_ as a function of the nanotube diameter for both families.

**Figure 3:**
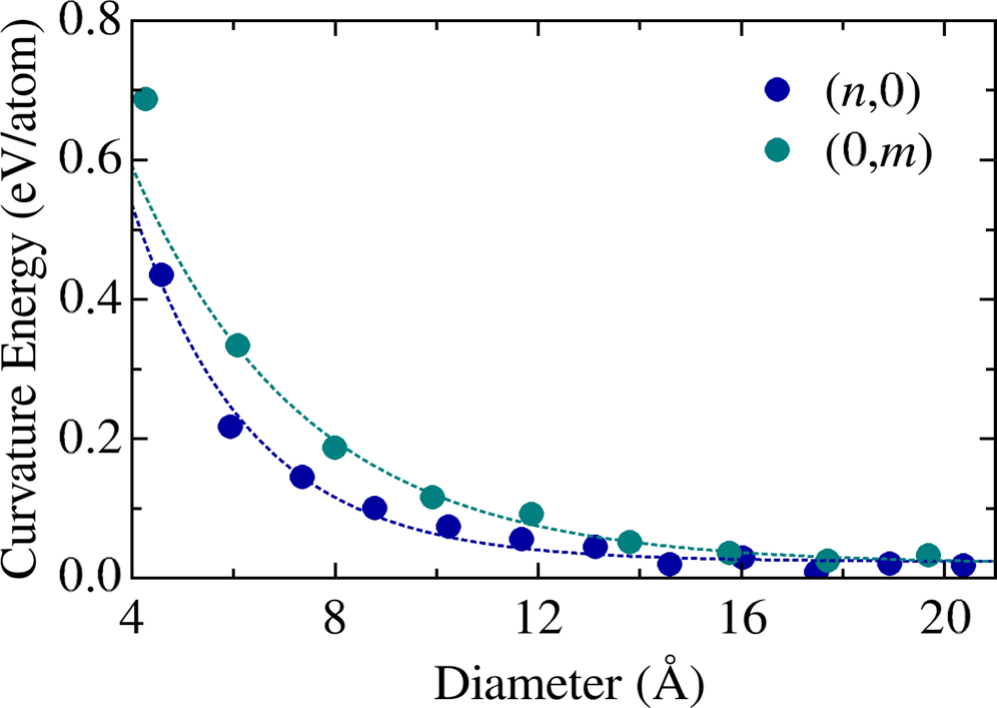
Curvature energy per atom as a function of TGNT diameter for the *(n,0)* and *(0,m)* families.

For small diameters, the *(n,0)* family exhibits greater energetic stability (i.e., lower *E*_*c*_) compared to the *(0,m)* family, indicating that rolling the TG sheet along the *x* direction requires less energy than along the *y* direction. This observation is consistent with the structural anisotropy of the TG lattice, as shown in Figure 1b, and arises from the different orientations of the tetragonal and hexagonal rings relative to the bending axis, which directly influence the elastic response. Note that the C_1_–C_1_ dimers lie along the periodic direction in the *(n,0)* TGNTs, being better accommodated in the nanotube geometry than in *(0,m)* TGNTs, where such bonds are orthogonal to the periodic direction.

Both curves follow a *E*_*c*_ ∝ 1/*R* ^2^ trend, where *R* = *D*/2 is the nanotube radius. The convergence of *E*_*c*_ toward zero for diameters *D* ≳ 16 Å, confirming that curvature effects become negligible for sufficiently large nanotubes, approaching the energetic stability of the flat 2D structure. These results reinforce the mechanical flexibility of TG and demonstrate its structural viability in cylindrical geometries across various curvature regimes.

It is important to mention that some aspects regarding the stability of tetragraphene have been previously considered in the literature. For instance, molecular dynamics simulations suggest that the thermal stability of the tetragraphene membrane is expected at temperatures at least up to 1000 K. Furthermore, phonon band structure calculations indicated the absence of imaginary modes, which is a necessary condition to assess the dynamic stability of the system.^25^ Finally, tetragraphene’s mechanical stability was tested under strain application^25,27^ with the structure being shown to maintain its integrity for large strain loads.

### 3.2 Mechanical Properties

To investigate the mechanical response of TG and its corresponding nanotubes, we computed stress–strain curves using the DFT framework under uniaxial tensile strain. Figure 4 presents the results for the TG sheet along the *x* and *y* directions, as well as for the TGNTs of the *(n,0)* and *(0,m)* types, corresponding respectively to horizontal and vertical rolling of the 2D sheet. In addition to Young’s modulus (*Y*), the curves allow extraction of the ultimate tensile strength (σ_*C*_), defined as the maximum stress value, and the fracture strain (*ε*_*C*_), corresponding to the structural failure point of the system.

**Figure 4:**
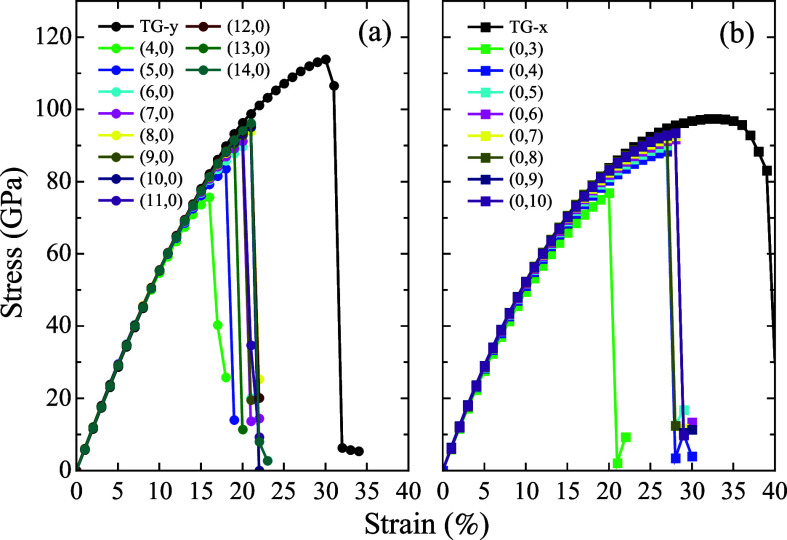
Stress–strain curves obtained for the TG and TGNT systems: (a) *(n,0)* TGNTs and TG under *y*-direction strain and (b) *(0,m)* TGNTs and TG under *x*-direction strain.

As expected, a general trend is observed in which increasing *n* or *m* leads to a convergence of the stress–strain curves of the TGNT, toward the 2D membrane behavior. This reflects the reduced impact of curvature effects, consistent with energetic and structural stability analyses previously discussed.

For a consistent comparison among the systems, all stress values are expressed in GPa. For the 2D TG sheet, we define the volume as the product of its *x* and *y* dimensions and an effective thickness *h* = τ + 3.35 Å, where 3.35 Å corresponds to the typical interlayer spacing in graphene, often adopted for carbon-based systems.^45^ For TGNTs, *h* is a function of τ, which depends on the diameter and is multiplied by the tube length *L* and its circumference D·π .

From Figure 4, it is evident that the Young’s modulus of TG is direction-dependent, ranging from approximately *Y* = 589 GPa along the *x*-axis to *Y* = 583 GPa along the *y*-axis. Notably, curvature introduces only minor variations in stiffness, with TGNTs exhibiting similar *Y* values. For the *(n,0)* chirality, Young’s modulus is even slightly higher for small diameters compared to the 2D case due to the alignment of the TG building blocks along the axial tensile direction, which increases stiffness in this specific direction (see Figure 1c). These values range from *Y* = 597.3 GPa (1.4% higher than in the planar case), for the (4,0) tube, to Y = 586 GPa (negligible difference), for (14,0), approaching the corresponding 2D value. Moving to the *(0,m)* configurations, curvature does not enhance rigidity. Here, the fundamental TG units are aligned perpendicularly to the tensile axis. Consequently, *Y* varies from 561.3 GPa (approximately 4% lower than the planar case), in the (0,3) tube, to 588 GPa, for the (10,0) case. In all studied tubes, the deviation from the 2D Young’s modulus remains within 20 GPa.

In contrast, the ultimate strength (σ_*C*_) and fracture strain (*ε*_*C*_) show strong sensitivity to curvature and tube diameter. For the TG sheet, σ_*C*_ reaches approximately 97 GPa and 114 GPa in the *x* and *y* directions, respectively, confirming the previously reported mechanical anisotropy, via reactive molecular dynamics simulations.^46^ For *(n,0)* TGNTs, σ_*C*_ increases with diameter from 75 GPa to 96 GPa, with significant changes only up to the (7,0) configuration, consistent with the recovery of the 2D-like properties for tubes wider than a certain threshold diameter value. The corresponding fracture strains decrease substantially, from ε_c_ = 34% in 2D, to values between 16% and 21% in nanotubes.

In the *(0,m)* family, the ultimate strength ranges from 76 GPa to 93 GPa, representing a reduction of nearly 20% compared to the 2D structure. However, their fracture strains are closer to the 2D reference, ranging from 20% to 28%. This reduction in strength and ductility in quasi-1D systems is primarily attributed to Poisson effects and the lower degrees of freedom available in nanotubes compared to planar systems. The elastic constants discussed here are summarized in Table 2.

**Table 2:** Elastic Parameters Obtained for the 2D TG Sheet and the TGNTs. Units: *Y* (GPa), *σ*_*C*_ (GPa), and *ε*_*C*_ (%)

TGs							
*x*	*Y*	σ_*C*_	*ε*_*C*_	*y*	*Y*	σ_*C*_	*ε*_*C*_
	589.44	97.29	34		583.74	113.90	30

TGNTs							
(*n,0*)	*Y*	σ_*C*_	*ε*_*C*_	(0, *m*)	*Y*	σ_*C*_	*ε*_*C*_
(4,0)	597.31	75.69	16	(0,3)	561.26	76.91	20
(5,0)	598.32	83.62	18	(0,4)	577.26	88.38	27
(6,0)	595.82	89.68	20	(0,5)	581.81	90.06	27
(7,0)	593.12	91.23	20	(0,6)	586.74	91.78	28
(8,0)	593.29	93.96	21	(0,7)	592.28	92.62	28
(9,0)	591.41	92.84	20	(0,8)	584.94	93.02	27
(10,0)	590.54	95.17	21	(0,9)	593.83	93.45	28
(11,0)	588.33	93.59	20	(0,10)	588.68	93.46	28
(12,0)	591.29	95.90	21				
(13,0)	586.80	91.44	19				
(14,0)	586.12	96.27	21				

To further illustrate the fracture mechanisms in TGNTs, we focus on the (12,0) and (0,9) configurations. These two particular tubes were chosen due to the good similarity between the radius of both tubes, corresponding to approximately 17 Å , and because they represent well the fracture/deformation pattern of other tubes in the two (n,0) and (0,m) system families. Figure 5 shows snapshots taken near the fracture point. As anticipated, the (12,0) TGNT undergoes a major structural transformation between 21% and 22% strain, resulting in a collapse into a new carbon-based structure devoid of sp^3^ hybridization. Otherwise, (0,9) TGNT exhibits a localized fracture with pronounced surface roughness and structural collapse between 28% and 29% strain. As discussed previously, these differences arise from the relative alignment of the C_1_–C_1_ bonds with respect to the tensile axis.

**Figure 5:**
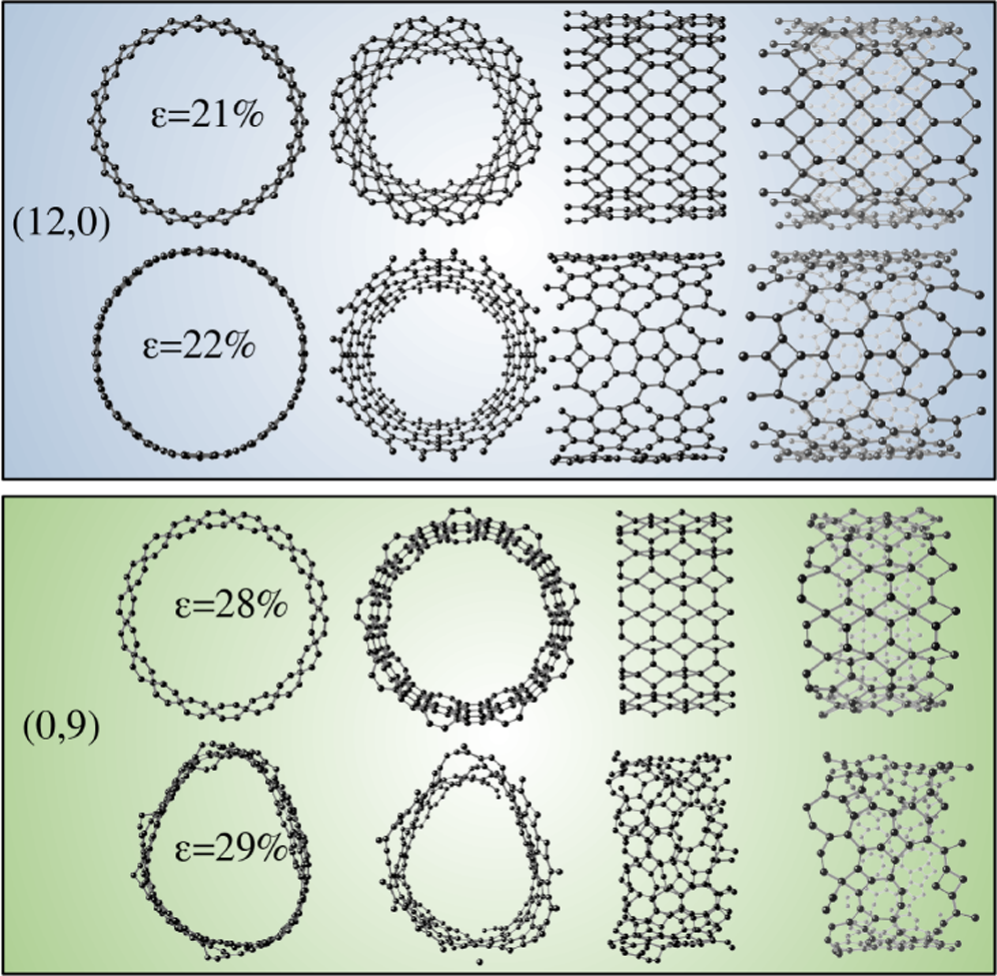
Orthogonal and perspective views of the fracture dynamics for TGNTs (12,0) and (0,9) at strains near rupture.

To give even more light to the fracture process, we show in Figure 6 the fracture dynamics of two particular nanotubes (14,0) and (0,10). The ring geometries of parts of the structures under strain are depicted in color in both panels. After reaching a 21% strain rate, the (14,0)-TGNT does not exhibit structural failure, which is observed at a later percentage, at 22% strain, where bond breaking and rebonding processes are seen. Other carbon rings, different from those found in the original structure, are observed. Similar behavior is verified for the (0,10)-TGNT; defects appear at 29% strain, while at 28% strain the nanotube maintains its original structural characteristic. This anisotropic character is remarkable even when considering nanotubes of similar diameters.

**Figure 6:**
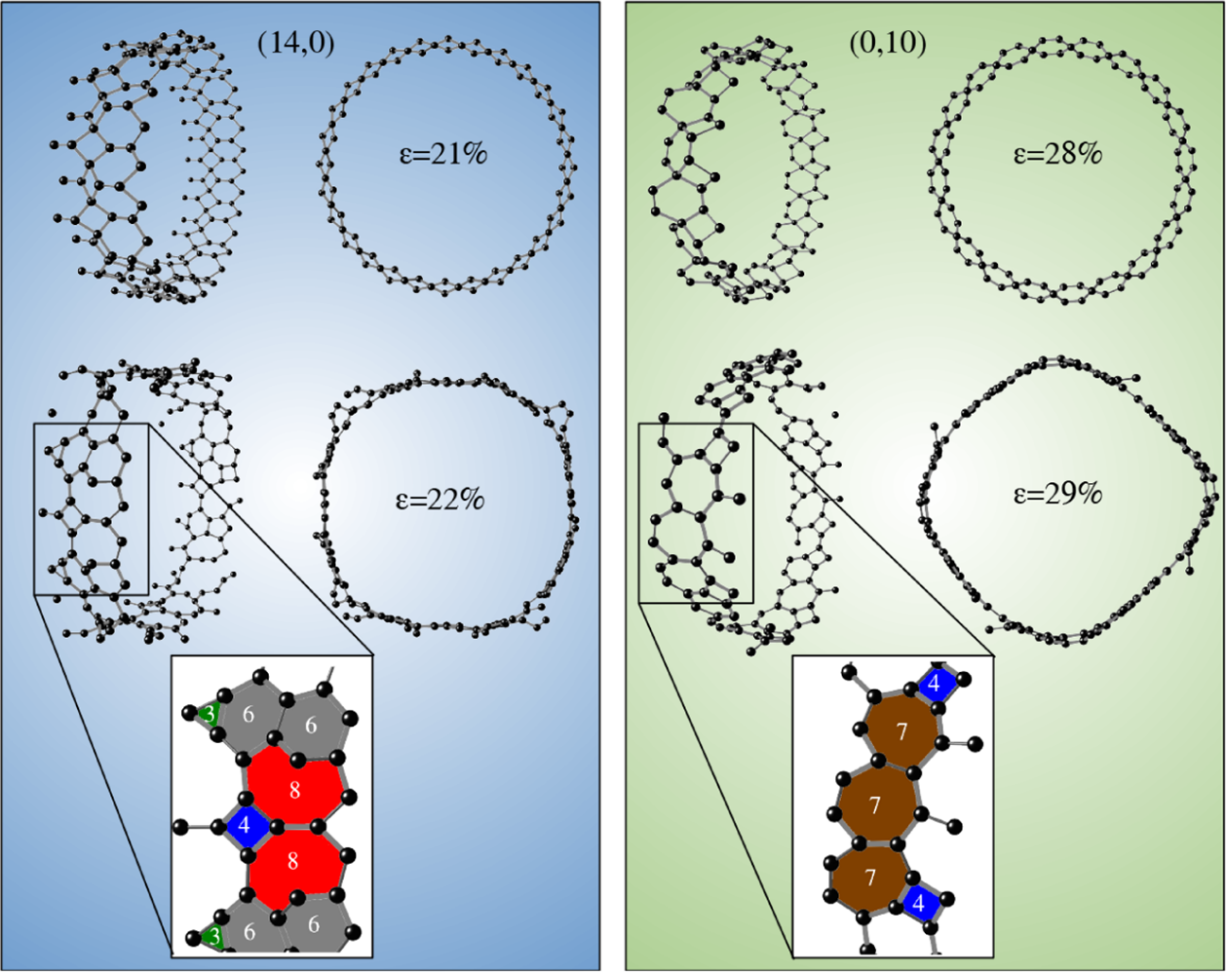
Structural fracture dynamics of (14,0) and (0,10) TGNTs. The ring geometries of part of the structures under strain are shown in color in the bottom parts.

We present in Figure 7 another aspect of describing the behavior of the membrane and nanotubes with the application of strain. The parabolic dependence of the strain energy with the rate is shown for the two studied TGNT families and the membrane. The structural collapse of (n,0) nanotubes is strongly influenced by the nanotube diameter, with the exception of the nanotube with the smallest diameter. As expected, with increasing diameters, the critical fracture points tend to gradually approach the critical point obtained for the membrane, shown in black curves.

**Figure 7:**
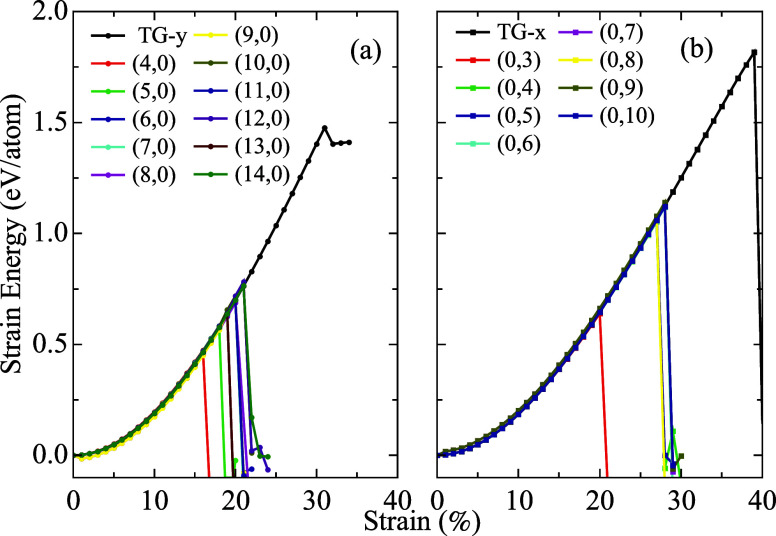
Strain energy versus strain for the two families of TGNTs and the TG membrane.

### 3.3 Electronic Properties

For electronic properties, we start revisiting the electronic structure of the 2D TG system, as it allows us to get qualitative insight into the electronic properties of TGNTs. In Figure 8a, we show the TG electronic band structure along the high-symmetry lines of the reciprocal space. As previously reported^25^ TG is semiconducting with both valence band maximum (VBM) and conduction band minimum (CBM) at the Γ point. However, the valence band is much more dispersive along the Γ – *X* path than along the Γ – *Y* line. This can be seen not only in Figure 8a, but also with the aid of Figure 8(b), where we plot the TG’s valence and conduction bands over the entire cell of the reciprocal space. On the other hand, the valence band shows further low dispersion along the *X-S* line, on the edge of the rectangular zone represented in Figure 8b. We observe that the conduction band is also more dispersive along Γ – *X* than along Γ – *Y* but with a lower difference than the one observed in the valence band. The bands are further degenerate two-by-two along the *X–S* and *S–Y* paths, since the rectangular setup is a conventional unit cell for TG.

**Figure 8:**
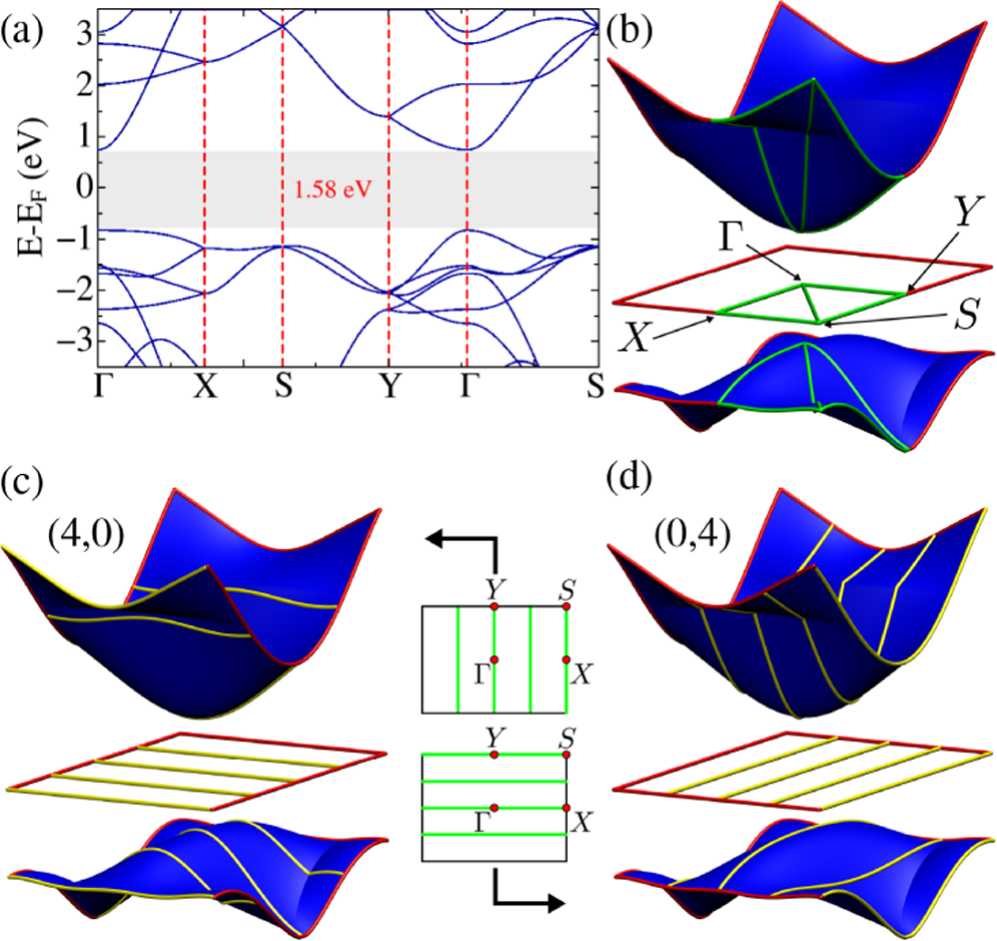
(a) Electronic band structure of TG represented along high-symmetry lines of the reciprocal space (the dotted green line at *E* = 0 represents the Fermi energy). (b) Surface plot over the entire reciprocal space cell for TG’s valence and conduction bands, with the Fermi energy represented by the horizontal red rectangle. The high-symmetry paths from (a) are here represented by green lines. (c) Representation of the cutting lines of a (4,0) TGNT represented over the reciprocal space cell (green lines) and over the surface plots for the valence and conduction bands (yellow lines). (d) Same as (c), but for the (0,4) TGNT.

In principle, any two-dimensional nanocarbon can be rolled up to form nanotube setups, and a reasonable understanding of their electronic structure can be inferred from the band structure of the parent 2D system. This is usually done through a zone-folding approach (ZF), where the nanotube bands can be approximated by the projection of the 2D band structure over a set of *k*-space cutting lines determined by the tube’s chirality. While this approach is usually known by its application to the graphene case,^47^ nanotubes based on other 2D nanocarbon can also be described by such a procedure.^48^

However, in TG-TGNT comparison, one expects ZF does not provide a quantitative good approach, unless one considers very wide nanotubes. Due to the trilayer structure of TG, the lower threshold diameter for a TGNT to be well described by ZF is expected to be much larger than for other one-atom-thick systems. Nevertheless, we can still obtain qualitative insight into the TGNT electronic structure through ZF. The cutting lines for a *(n,0)* nanotube will always be parallel to the Γ – *Y* path from TG’s reciprocal space. In contrast, *(0,m)* TGNTs have cutting lines parallel to the Γ – *X* direction of the 2D system. In Figure 8c, we illustrate the cutting lines of a (4,0) nanotube over the cell of the reciprocal space and over the surface plots for TG’s valence and conduction bands. As any *(n,0)* TGNT, (4,0) has a line passing by the Γ – *Y* line, so that we expect a dispersive character for both valence and conduction bands. The (4,0) case also has a feature shared only with *(n,0)* tubes with even *n*: a cutting line passing along *X-S*. For such a line, the valence band is low-dispersive, and a similar occupied nanotube band is expected for even *n* cases. In Figure 8d we illustrate the same for the (0,4) case. The central cutting line passing through Γ – *X* (presented in any tube with the same chirality) results in a low dispersive profile for the valence band, and a much more dispersive character for the conduction branch.

In the following, the electronic properties of TGNTs were investigated through a first-principles DFT-based framework. Figure 9 presents the electronic band structures obtained for TGNTs of the *(n,0)* and *(0,m)* families, considering different diameters. The results show that all configurations exhibit semiconducting behavior, with band gap values depending on the chirality and tube diameter.

**Figure 9:**
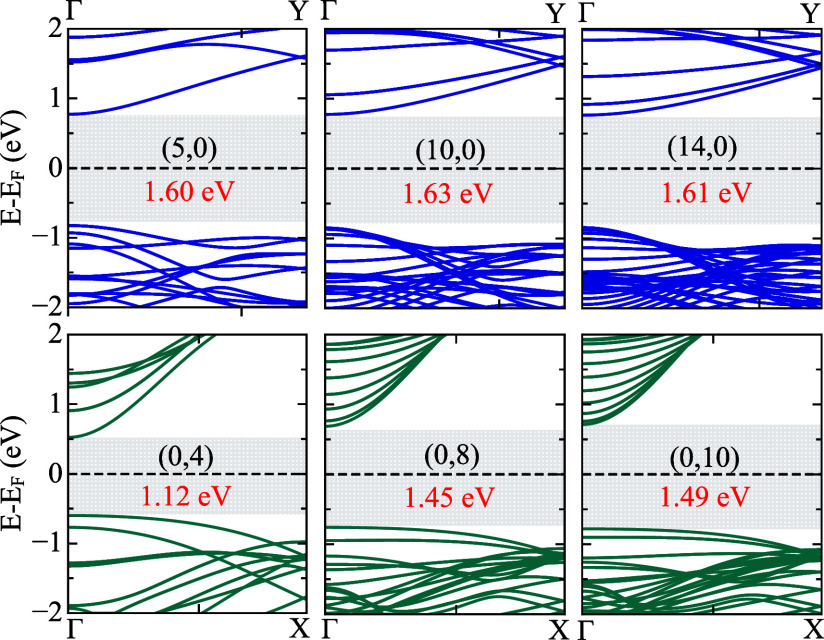
Electronic band structures for tetragraphene nanotubes of the *(n,0)* (top panels) and *(0,m)* (bottom panels) families. Shaded areas indicate the band gaps, with values in red.

All configurations display direct band gaps at the Γ point. Such a feature remains robust under diameter variation and changes in chirality, including structures whose band structures were not shown in Figure 9, suggesting the preservation of the underlying electronic topology of the rolled-up tetragraphene sheet in the cylindrical setup. The preservation of this direct gap supports potential applications in nanoscale optoelectronic devices, such as light emitters and photodetectors. In addition, qualitative aspects of the bands can be understood from a comparison with the 2D system, based on the ZF approach. The *(n,0)* tubes have a dispersive valence band (originating from TG’s Γ – *Y* path) that crosses other less dispersive valence bands (originating from TG’s *X-S* path or other close lines), while the conduction band shows a slightly less dispersive character. These aspects can be seen by comparing the upper panel from Figures 9 with [Fig fig8]c. For *(0,m)* tubes, we have a low dispersive valence band (especially around the Γ point) which can be associated with the TG’s Γ – *X* line (see Figure 8d), with the conduction band having a much more pronounced dispersion. However, we note that curvature plays a non-negligible role, as the TGNTs band gap values are significantly different from those of the 2D counterpart.

From the band structures, it is further possible to infer the effective mass of charge carriers qualitatively. In *(n,0)* TGNTs, the conduction bands near the Fermi level are relatively flat, indicating a higher effective electron mass. In contrast, *(0,m)* nanotubes exhibit more dispersive conduction bands, suggesting lower effective masses and higher charge mobility. This contrast is consistent with the relative orientation of the tetragraphene building blocks along the tube axis and the electronic structure of the TG layer, as discussed above. In the *(0,m)* configuration, the axial direction coincides with the *x* direction of the 2D sheet, which is characterized by a higher electronic dispersion. In the *(n,0)* configuration, the axial direction aligns with the *y* direction, which is less favorable for electronic transport, increasing confinement effects and the effective mass. Moreover, weakly dispersive (flat) bands are observed below the Fermi level, particularly in the *(n,0)* tubes. These bands may correspond to localized states that do not contribute significantly to conduction but could be relevant in optical absorption or doping scenarios. From a ZF point of view, the localized valence bands of *(n,0)* are related to cutting lines close to the *X-S* edge of the reciprocal space cell. These differences between the *(n,0)* and *(0,m)* setups can be further connected to structural features of TG and the chiralities of the nanotubes. In the *(n,0)* cases, the lines of tetragons (linked to each other by *sp* ^3^ atoms) form rings around the tube circumference, separating the strips of contiguous hexagons (graphitic-like sector) from each other and resulting in less dispersive bands. For the *(0,m)* cases, the strips of connected hexagons lie along the tube’s periodic direction, favoring delocalization along the tube axis.

Figure 10 summarizes the dependence of the energy band gap on the nanotube diameter. For the *(n,0)* family, the gap remains nearly constant at around 1.6 eV, even for small-diameter tubes. This indicates that the curvature induced by zigzag-like rolling has little effect on frontier electronic states. This behavior closely follows the gap value of the flat 2D sheet, indicated by the dashed red line.

**Figure 10:**
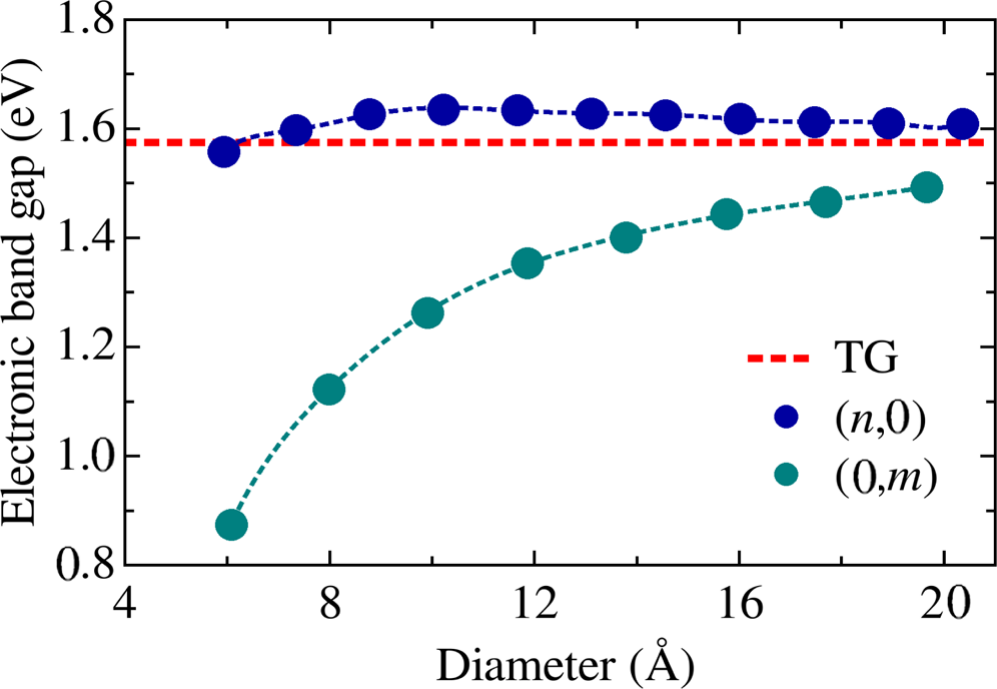
Electronic band gap as a function of nanotube diameter for both *(n,0)* and *(0,m)* TGNTs. The dashed red line corresponds to the band gap of 2D tetragraphene.

In contrast, *(0,m)* nanotubes exhibit a marked increase in the band gap with increasing diameter, changing by approximately 0.6 eV from narrow to wider tubes. This indicates that armchair-like rolling allows for more sensitive tuning of the energy separation between the valence and conduction bands. We further note that the difference between a TGNT band gap and the 2D TG band gap is much larger for the *(0,m)* tubes compared to the *(n,0)* counterparts. This higher sensitivity of *(0,m)* TGNTs to curvature can also be associated with structural details. Note that the C_1_–C_1_ bonds are orthogonal to the periodic direction in the *(0,m)* systems, so that their bond length is more affected by curvature than in *(n,0)* tubes, as we discussed before on the data from Figure 2. Such C_1_–C_1_ bonds are those that involve *sp* ^2^-like atoms, those with the most relevant states for the frontier states, since they have a π-like state. Differently, the C_2_ atoms bind to other atoms exclusively by strong σ states, participating less in frontier levels, and resulting in more localized states. This is consistent with the spatial distribution of TG’s frontier states as shown in the previous literature.^25,26^

These findings highlight the potential of TGNTs for structural band gap engineering via topological parameters such as chirality and diameter. The tunable gap range, spanning from 1.0 to 1.6 eV, covers a significant portion of the visible and near-infrared spectrum. Notably, this modulation does not require chemical functionalization or external fields, making TGNTs promising candidates for tunable optoelectronic devices, photonic sensors, and flexible electronics.

All gap values were obtained using the PBE functional, which systematically underestimates absolute band gaps. Nevertheless, the use of PBE is justified by its favorable balance between accuracy and computational efficiency, especially in light of the large number of structures and deformation conditions explored. As our primary focus lies on comparative trends and qualitative behavior, more accurate gap values obtained with hybrid functionals, such as HSE06 ^49,50^ serve only as a reference. Previous results for 2D tetragraphene, using HSE06, report gaps on the order of 3.7 eV.^25^

The influence of uniaxial strain on the electronic properties of both TGNTs and the 2D tetragraphene sheet was also investigated. Figure 11 displays the evolution of the band gap, as a function of applied strain, considering strain along the *x* and *y* directions for the 2D sheet and axial strain for the nanotubes. The strain response exhibits highly anisotropic and chirality-dependent behavior. We should mention that for some tubes, no points are expected in the curves presented in Figure 11 in the strain range of 20 and 30%, due to the fact that those tubes have already broken under such strain values. For *(n,0)* TGNTs, as well as for the 2D sheet strained along the *y* direction, the band gap initially increases with strain, reaching a maximum near 8%, followed by a sharp decrease leading to complete gap closure around 18–20%. This transition to a metallic state does not result from immediate structural failure but rather from a strain-induced reorganization of the electronic structure, which brings the conduction band states closer to the Fermi level.

**Figure 11:**
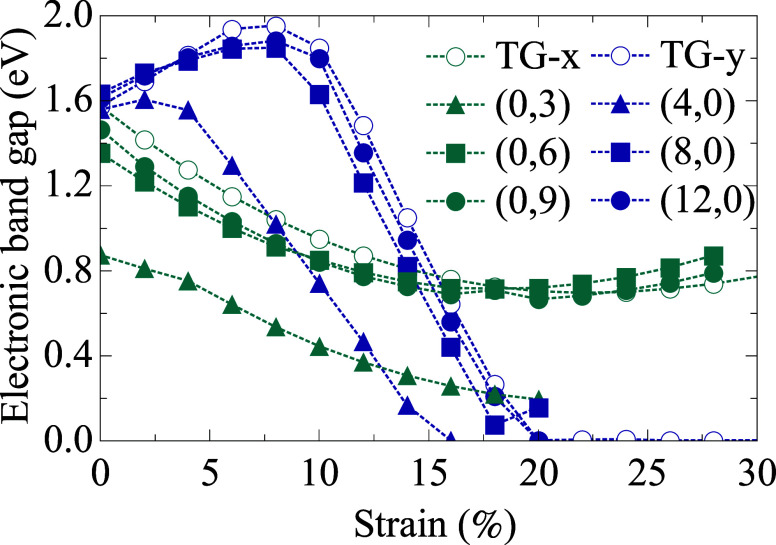
Electronic band gap as a function of applied strain. Open symbols represent the 2D TG sheet, while filled symbols correspond to TGNTs.

To understand this gap-closing mechanism, we look at the spatial distribution of the local density of states (LDOS) over the valence band maximum (VBM) and conduction band minimum (CBM) of the TG sheet, since this semiconductor-to-metal transition occurs for TG and all the *(n,0)* tubes at similar strain values. In Figure 12(a,b) we show the LDOS plots for the VBM and CBM of TG for the relaxed structure (0%) and for several strain values along *y*, as well as their corresponding electronic band structures in Figure 12c. Strain along *y* was chosen since it is for strain along this direction that TG undergoes the semiconductor-to-metal transition, as well as the periodic direction of the *(n,0)* tubes can be projected along this direction of the parent sheet. For the strain-free structure, the LDOS for the frontier states is particularly distributed over π-like orbitals on the tricoordinated carbon atoms of TG. This pattern is overall maintained for the CBM up to 10% strain (see Figure 12a). For 12% strain, the electronic cloud of the CBM moves to the central plane, below the tri coordinated atoms, maintaining this character until 18% strain. Looking at the band structures at Γ, displayed in Figure 12c, we note a branch above the conduction band that moves toward the Fermi energy as we increase strain from 0 to 12% strain. This upper band reaches the conduction band at around 10% strain and crosses it, becoming the system’s new conduction band at 12% strain. This is why we observe the change in the CBM pattern from 10% to 12% strain. For the VBM (see Figure 12b), the LDOS also changes its pattern as strain evolves from 4% to 8%, becoming more pronounced in the central plane of the system. It turns out that the energy value of the valence band at Γ (the VBM of the relaxed case) shifts down with strain, so that the VBM becomes closer to the *X* point of the BZ (but over the same band). In addition, a dispersive branch below the valence band shifts up, at Γ, with increasing strain (see the band structures in Figure 12c. This band crosses the original valence band of the system from 6% to 8% strain, and becomes the global VBM as we go from 10% to 12% strain, where we observe a new change in the VBM pattern. For strain above 12% , the VBM coming from this new valence band is more related to the tetra coordinated atoms. The larger participation of sp^3^ atoms in the TG’s frontier states is not surprising, as the four bonds from the atoms in the central plane of the sheet become closer to a planar configuration as strain increases. With the bonds approaching each other, this results in a metastable configuration for the sp^3^ hybridized carbons. The instability brought up by such structural configuration brings the levels associated with the σ-like bonds from these tetra coordinated atoms closer to the Fermi energy. For 20% and 22% strain, these new valence/conduction bands (promoted by strain) cross each other, closing the system’s gap. Plotting the VBM and CBM of TG for these strain rates shows that these are mostly σ states that originate from the tetra-coordinated atoms. In other words, strain brings the sp^3^ bonds close to each other, shifting their respective frontier states toward the Fermi level until the point the frontier bands cross each other for 20% strain.

**Figure 12:**
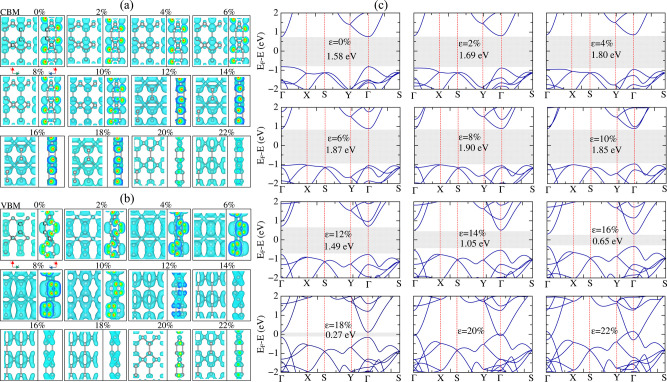
Local density of states plots for the (a) CBM and (b) VBM levels at TG under different values of strain applied in the *y*-direction. For each strain value, we show both upper and side views. (c) Electronic band structures of TG for the same strain configurations from (a-b). The energy gaps are highlighted by gray strips in each panel, together with the gap and strain values.

This finding is particularly novel for tetragraphene and demonstrates that a strain-driven semiconductor-to-metal transition can occur while preserving the underlying sp^2^-sp^3^ hybridization. The emergence of a stable metallic state under uniaxial strain expands the potential of TGNTs in mechanically responsive electronic applications.

In contrast, *(0,m)* TGNTs and the 2D sheet strained along the *x* direction exhibit a smoother and more gradual decrease in the band gap, stabilizing around 0.8 eV, even under strains surpassing 25%. This indicates that the coupling between frontier electronic states is less favorable in this orientation, maintaining the semiconducting nature of the material along the entire strain range considered.

The results demonstrate that uniaxial strain, combined with chirality and diameter, provides an effective mechanism for tailoring the electronic properties of TGNTs. Different setups of graphene-based optical fiber sensors have been reported in recent years, incorporating graphene to enhance sensitivity to strain.^51^ In particular, high-performance and low-cost CNT photodetectors have been proposed as a great potential for future high-speed optical communication.^52^ Other carbon-based materials, such as biphenylene, were proposed to promote tuning of an anisotropic electronic current in monolayers, mediated by external mechanical strain, as smart applications in nanoelectronic devices.^53^ Our findings indicate that the observed versatility underscores the potential of these TTG and TGNT nanostructures for strain-sensitive optoelectronic devices, with reversible phase transitions and a wide range of tunable electronic behavior.

## 4 Conclusions

In this study, we systematically investigated the structural, mechanical, and electronic properties of tetragraphene nanotubes (TGNTs) using density functional theory. By analyzing two primary chirality families-*(n,0)* (zigzag-like) and *(0,m)* (armchair-like)-we demonstrated that the curvature introduced by the TGNT setups does not compromise the semiconducting nature of tetragraphene, and that all TGNTs maintain direct band gaps located at the Γ point.

The electronic properties were found to be strongly dependent on chirality and diameter. While *(n,0)* TGNTs exhibit nearly constant band gaps close to those of the 2D sheet, *(0,m)* nanotubes exhibit an increasing band gap with diameter, allowing precise modulation in the visible and near-infrared spectrum. This band gap tunability, achieved without chemical functionalization or external fields, establishes TGNTs as promising materials for optoelectronic devices that require tunable electronic responses.

Strain engineering also revealed rich and unprecedented behavior. For the first time, we report a strain-induced semiconductor-to-metal transition in TG-based systems, occurring in *(n,0)* TGNTs at approximately 20% axial strain. This transition occurs without structural collapse or loss of hybridization, indicating a purely electronic reconfiguration. Conversely, *(0,m)* nanotubes remain semiconducting under strain, with smoother gap reduction profiles. Mechanically, TGNTs exhibit high stiffness values and distinctive anisotropic responses to deformation. The structural integrity of the sp^2^-sp^3^ hybrid network remains intact under curvature and strain, underscoring the mechanical robustness of these nanostructures. Together, our findings establish that topological design and mechanical strain are effective and complementary strategies for tuning the electronic and mechanical responses of tetragraphene-based systems. The ability to induce reversible phase transitions, combined with their flexibility and robustness, positions TGNTs as versatile building blocks for strain-sensitive, flexible nanoelectronic and optoelectronic applications.

## References

[1] Lei Y., Zhang T., Lin Y.C., Granzier-Nakajima T., Bepete G., Kowalczyk D.A., Lin Z., Zhou D., Schranghamer T.F., Dodda A. (2022). Graphene and Beyond:
Recent Advances in Two-Dimensional Materials Synthesis, Properties,
and Devices. ACS Nano.

[2] Hosseini M., Soleimani M., Shojaei F., Pourfath M. (2024). Graphsene as a novel
porous two-dimensional carbon material for enhanced oxygen reduction
electrocatalysis. Sci. Rep..

[3] Hong I., Bae H., Ahn J., Shin H., Lee H., Kwon Y. (2024). Design of
biphenylene-derived tunable dirac materials. FlatChem.

[4] Alves R. A. F. L., Lima K. A., da Silva D. A., Mendonça F. L. L., Ribeiro Junior L. A., Pereira Junior M. L. (2025). Computational
Design of 2D Nanoporous Graphene via Carbon-Bridged Lateral Heterojunctions
in Armchair Graphene Nanoribbons. ACS Omega.

[5] Novoselov K. S., Geim A. K., Morozov S. V., Jiang D.-E., Zhang Y., Dubonos S. V., Grigorieva I. V., Firsov A. A. (2004). Electric field effect
in atomically thin carbon films. Science.

[6] Zorn N. F., Zaumseila J. (2021). Charge transport
in semiconducting carbon nanotube
networks. Appl. Phys. Rev..

[7] Allard C., Alvarez L., Bantignies J.-L., Bendiab N., Cambré S., Campidelli S. E., Fagan J. A., Flahaut E., Flavel B., Fossard F. (2024). Advanced 1D heterostructures based on nanotube templates
and molecules. Chem. Soc. Rev..

[8] Sagade A. A., Nyayadhish A. (2020). A carbon nanotube–graphene nanoribbon seamless
junction transistor. Nanoscale Adv..

[9] Uddin M. M., Kabira M. H., AliOrcid M. A., Hossain M. M., Khandaker M. U., Sumit Mandald A. A., Jana D. (2023). Graphene-like emerging 2D materials:
recent progress, challenges and future outlook. RSC Adv..

[10] Fan Q., Yan L., Tripp M., Krejci O., Dimosthenous S., Kachel S., Chen M., Foster A., Koert U., Liljeroth P., Gottfried J. (2021). Biphenylene network: A nonbenzenoid
carbon allotrope. Science.

[11] Lage L. L., Arroyo-Gascón O., Chico L., Latgé A. (2024). Robustness
of type-II Dirac cones in biphenylene: From nanoribbons to symmetric
bilayer stacking. Phys. Rev. B.

[12] López-Alcalá D., Baldovı J. J. (2025). Magnetic
proximity effect in biphenylene monolayer
from first-principles. J. Mater. Chem. C.

[13] Hou L., Cui X., Guan B., Wang S., Li R., Liu Y., Zhu D., Zheng J. (2022). Synthesis of a monolayer fullerene network. Nature.

[14] Li Q., Li Y., Chen Y., Wu L., Yang C., Cui X. (2018). Synthesis
of *γ*-graphyne by mechanochemistry and its electronic
structure. Carbon.

[15] Rodrigues C. M., Lage D. L., Venezuela L., Latge P. A. (2022). Exploring the enhancement
of the thermoelectric properties of bilayer graphyne nanoribbons. Phys. Chem. Chem. Phys..

[16] Rodrigues D. C. M., Amorim R. G., Latgé A., Venezuela P. (2023). Improving
the sensitivity of graphyne nanosensor by transition metal doping. Carbon.

[17] Zhang S., Zhou J., Wang Q., Chen X., Kawazoe Y., Jena P. (2015). Penta-graphene: A new carbon allotrope. Proc.
Natl. Acad. Sci. U. S. A..

[18] Girão E. C., Macmillan A., Meunier V. (2023). Classification of sp2-bonded carbon
allotropes in two dimensions. Carbon.

[19] Dos
Santos R., Sousa L., Galvao D., Ribeiro L. (2020). Tuning Penta-Graphene
Electronic Properties Through Engineered Line Defects. Sci. Rep..

[20] Yuan P. F., Zhang Z. H., Fan Z. Q., Qiu M. (2017). Electronic structure
and magnetic properties of penta-graphene nanoribbons. Phys. Chem. Chem. Phys..

[21] De
Sousa J., Aguiar A., Girão E., Fonseca A. F., Coluci V., Galvão D. (2021). Mechanical
properties of single-walled penta-graphene-based nanotubes: A DFT
and Classical molecular dynamics study. Chem.
Phys..

[22] Zhang X., Wei L., Tan J., Zhao M. (2016). Prediction of an ultrasoft graphene
allotrope with Dirac cones. Carbon.

[23] Deng Y.-X., Chen S.-Z., Zeng Y., Feng Y., Zhou W.-X., Tang L.-M., Chen K.-Q. (2018). Spin gapless
semiconductor and half-metal
properties in magnetic penta-hexa-graphene nanotubes. Org. Electron..

[24] Deng Y.-X., Chen S.-Z., Zhang Y., Yu X., Xie Z.-X., Tang L.-M., Chen K.-Q. (2020). Penta-hexa-graphene
nanoribbons:
intrinsic magnetism and edge effect induce spin-gapless semiconducting
and half-metallic properties. ACS Appl. Mater.
Interfaces.

[25] Ram B., Mizuseki H. (2018). Tetrahexcarbon:
A two-dimensional allotrope of carbon. Carbon.

[26] de
Vasconcelos F. M., Souza Filho A. G., Meunier V., Girão E. C. (2020). Electronic
and structural properties of tetragraphenes. Carbon.

[27] Wei Q., Yang G., Peng X. (2020). Auxetic Tetrahex Carbon with Ultrahigh
Strength and a Direct Band Gap. Phys. Rev. Appl..

[28] Hoat D., Amirian S., Alborznia H., Laref A., Reshak A., Naseri M. (2021). Strain effect on the
electronic and optical properties
of 2D Tetrahexcarbon: a DFT-based study. Indian
J. Phys..

[29] Ma S., Zhang H., Cheng Z., Xie X., Zhang X., Liu G., Chen G. (2024). A novel Tetrahexcarbon as a high-performance anode
material for Na-ion and K-ion batteries. Colloids
Surf., A.

[30] Chaves A., Azadani J.G., Alsalman H., Da Costa D.R., Frisenda R., Chaves A.J., Song S.H., Kim Y.D., He D., Zhou J. (2020). Bandgap
engineering of two-dimensional semiconductor
materials. Npj 2D Mater. Appl..

[31] Foa Torres, L. E. F. ; Roche, S. ; Charlier, J.-C.-C. Introduction to Graphene-Based Nanomaterials From Electronic Structure to Quantum Transport. 2nd ed.; 2021, Cambridge University Press ,452. 10.1557/mrs.2015.109.

[32] Torres V., Faria D., Latgé A. (2021). Switching valley filtered current
directions in multiterminal graphene systems. Phys. Rev. B.

[33] Mahmud M. T., Zhai D., Sandler N. (2023). Topological flat bands in strained
graphene: Substrate engineering and optical control. Nano Lett..

[34] Kilic M. E., Lee K.-R. (2020). Tuning the electronic, mechanical,
thermal, and optical
properties of tetrahexcarbon via hydrogenation. Carbon.

[35] Kilic M. E., Lee K.-R. (2020). First-Principles Study of Fluorinated Tetrahexcarbon:
Stable Configurations, Thermal, Mechanical, and Electronic Properties. J. Phys. Chem. C.

[36] de
Vasconcelos F. M., Souza Filho A. G., Meunier V., Girão E. C. (2019). Electronic
properties of tetragraphene nanoribbons. Phys.
Rev. Mater..

[37] Yang G., Yang Y., Peng X. (2020). Systematic
theoretical study of carbon
nanotubes rolled from a two-dimensional tetrahex-carbon nanosheet. Phys. Rev. B.

[38] Silva P. V., Lamparski M., Aguiar A. L., Souza Filho A. G., Meunier V., Girão E. C. (2021). Structural and electronic properties
of double-walled *α*-graphyne nanotubes. Comput. Mater. Sci..

[39] Silva P. V., Girão E. C. (2023). Structural and electronic properties of collapsed armchair
single-walled *α*-graphyne nanotubes. Comput. Mater. Sci..

[40] Soler J. M., Artacho E., Gale J. D., García A., Junquera J., Ordejón P., Sánchez-Portal D. (2002). The SIESTA
method for ab initio order-N materials simulation. J. Phys.: condens. Matter..

[41] Perdew J. P., Burke K., Ernzerhof M. (1996). Generalized
Gradient Approximation
Made Simple. Phys. Rev. Lett..

[42] Troullier N., Martins J. L. (1991). Efficient pseudopotentials
for plane-wave calculations. Phys. Rev. B.

[43] Anglada E., Soler M. J., Junquera J., Artacho E. (2002). Systematic generation
of finite-range atomic basis sets for linear-scaling calculations. Phys. Rev. B.

[44] Monkhorst H. J., Pack J. D. (1976). Special points for
Brillouin-zone integrations. Phys. Rev. B.

[45] Hess P. (2020). Thickness
of elemental and binary single atomic monolayers. Nanoscale Horiz..

[46] Brandão W. H., Aguiar A. L., Fonseca A. F., Galvão D., De Sousa J. (2023). Mechanical properties of tetragraphene single-layer:
A molecular dynamics study. Mech. Mater..

[47] Barone V., Scuseria G. E. (2004). Theoretical study
of the electronic properties of narrow
single-walled carbon nanotubes: Beyond the local density approximation. J. Chem. Phys..

[48] Silva P. V., Souza Filho A. G., Meunier V., Girao E. C. (2019). Structural and electronic
properties of nanotubes constructed from fragmented fullerenes. Carbon.

[49] Heyd J., Scuseria G. E. (2004). Efficient hybrid
density functional calculations in
solids: Assessment of the Heyd–Scuseria–Ernzerhof screened
Coulomb hybrid functional. J. Chem. Phys..

[50] Hummer K., Harl J., Kresse G. (2009). Heyd-Scuseria-Ernzerhof
hybrid functional
for calculating the lattice dynamics of semiconductors. Phys. Rev. B.

[51] Miguel H., Zamarreño C. R., Melendi-Espina S., Bird L., Mayes A. G. (2025). Optical
Fibre Sensors Using Graphene-Based Materials: A Review. Sensors.

[52] Wu W., Ma H., Cai X., Han B. E. (2023). High-Speed Carbon Nanotube Photodetectors
for 2 m Communications. ACS Nano.

[53] Kuritza D. P., Miwa R. H., Padilha J. E. (2024). Directional dependence of the electronic
and transport properties of biphenylene under strain conditions. Phys. Chem. Chem. Phys..

